# Hook plate versus locking plate fixation in lateral clavicle fractures: operative efficiency and cumulative surgical burden in a propensity score–matched cohort study

**DOI:** 10.1007/s00068-026-03201-9

**Published:** 2026-04-27

**Authors:** Désirée Schibler, Michelle Antonia Hinkelmann, Flurina Frei, Hans-Christoph Pape, Florin Allemann, Christiane Barthel

**Affiliations:** 1 Department of Traumatology, University Hospital Zurich, Zurich, Switzerland; 2 University of Zurich, Zurich, Switzerland; 3 Department of Shoulder and Elbow Surgery, Schulthess Klinik, Lengghalde 2, Zurich, CH-8008 Switzerland

**Keywords:** Hook plate, Lateral clavicle fracture, Operative time, Implant removal, Surgical burden, Rropensity score matching

## Abstract

**Introduction:**

Hook plates remain controversial in the management of lateral clavicle fractures despite their biomechanical advantages. Concerns include implant-related complications, subacromial irritation, and the need for routine implant removal. The aim of this study was to compare operative efficiency and surgical burden of hook plate fixation with locking plate fixation in lateral clavicle fractures.

**Methods:**

A retrospective cohort analysis was conducted at a single tertiary trauma center between January 1, 2016, and December 31, 2024. Surgically treated lateral clavicle fractures were identified. Propensity score matching was performed based on age, sex, and AO fracture classification to reduce confounding, and matched pairs were compared between hook plate fixation (Group HP) and locking plate fixation (Group LCP). Outcome measures included operative time, complications, implant removal, radiographic callus formation, and refracture rates.

**Results:**

A total of 101 lateral clavicle fractures were identified. After propensity score matching, 44 matched pairs (Group HP vs. Group LCP) were analyzed, with comparable baseline characteristics between groups. Operative time was significantly shorter in Group HP (77.8 ± 28.4 vs. 93.5 ± 30.2 min, p = 0.018). Complication rates were higher in Group HP but did not reach statistical significance (18.2% vs. 4.5%, p = 0.093). Implant removal was performed more frequently in Group HP (100% vs. 25%, p < 0.001), although removal procedures were shorter (37.7 ± 21.5 vs. 65.7 ± 40.3 min, p = 0.003). Refracture rates after implant removal did not differ between groups, and no significant difference in radiographic callus formation was observed.

**Conclusion:**

In this propensity score–matched cohort, hook plate fixation was associated with shorter primary operative time but required routine implant removal, resulting in increased cumulative operative exposure. No significant differences in complications or radiographic callus formation were observed. Hook plates may be a useful option in selected fractures, but the need for implant removal must be considered. Further studies including functional outcomes are needed.

**Level of evidence:**

Level III, Retrospective comparative study.

## Introduction

Lateral clavicular fractures account for approximately 15–20% of all clavicular fractures and the lateral fixation can be challenging [1–4]. The acromioclavicular joint, coracoclavicular ligaments, and limited bone stock in the distal fragment are prone to secondary complications [5, 6]. As nonunion rates of up to 33% were described after nonoperative management, various fixation methods have been proposed to address the issues raised above [7–9]. While a variety of techniques have been described, most contemporary studies report that hook plate fixation and angle-stable locking plate fixation, with or without coracoclavicular augmentation, represent the most commonly used surgical strategies in current clinical practice [10–12].

The first implant that tried to address these disadvantages was the hook plate through subacromial fixation [13]. Hook plates are widely used due to their mechanical advantage in stabilizing small distal fragments and maintaining acromioclavicular joint biomechanics [14, 15].

Despite widespread clinical use, systematic evaluation of hook plate performance has yielded conflicting results and the necessity of hardware removal was criticized [16, 17]. Concerns have also been raised regarding subacromial impingement, rotator cuff damage, peri-implant fractures, and technical difficulties during implant removal [18, 19].

This has led to the development of various lateral implants, especially since locked plating has become available [1, 7].

The primary objective of this study was to evaluate operative efficiency and cumulative surgical burden of hook plates compared to locking plate fixation in lateral clavicle fractures, with particular focus on operative time and perioperative parameters.

## Methods

### Study design and setting

Patients treated at our hospital between January 1, 2016, and December 31, 2024, for lateral clavicle fractures were retrospectively identified. Included were patients aged 16 years or older who underwent operative fixation and had at least one postoperative radiographic follow-up at a minimum of 6 weeks postoperatively. Patients treated nonoperatively or with incomplete follow-up (e.g., no postoperative imaging or follow-up at another institution) were excluded. The study was approved by the local ethics committee, and all patients had provided institutional general consent allowing the use of anonymized clinical data for research purposes.

### Data collection and variables

Demographic and procedural data, including age, sex, side of injury, implant type, and surgical details, were extracted from the institutional information system. Fractures were classified according to the AO/OTA classification, and the number of fracture fragments was recorded.

The type of fixation (hook plate or locking plate) was documented. The choice of implant was at the discretion of the treating surgeon, based on individual clinical judgment and experience, as no standardized treatment protocol was in place. Hook plate fixation was performed using the VA-LCP™ Clavicle Hook Plate System (DePuy Synthes, Johnson & Johnson), whereas locking plate fixation was performed using standard VA-LCP plates (DePuy Synthes, Johnson & Johnson). Additional interfragmentary screw fixation was used in selected cases. Procedures were performed by different trauma surgeons of the department according to institutional standards. All procedures were performed with the patient in the beach chair position. Postoperative management consisted of early mobilization within the pain-free range of motion without routine immobilization. Abduction was limited to 90° during the early postoperative phase in both groups. Weight-bearing and loading of the affected arm were restricted for 6 weeks in both groups.

Operative time for both primary osteosynthesis and hardware removal was defined as the interval between skin incision and final skin closure, as documented in the anesthesia records. Data on postoperative follow-up, complications, and reoperations were obtained from clinical and radiographic documentation. Recorded complications included infection, osteolysis, material failure, peri-implant fracture, pseudoarthrosis, and clinically relevant pain.

Radiographic callus formation was evaluated on conventional follow-up radiographs and categorized as early (Group EC ≤ 6 weeks), intermediate (Group IMC > 6–26 weeks), late (Group LC > 26 weeks), or none (no visible callus, Group NC).

Radiographs were reviewed independently by three trauma surgeons. Inter-rater reliability was assessed by three trauma surgeons evaluating 151 radiographs for callus formation using a six-category scale (Fleiss’ κ). Intra-rater reliability was determined by repeat assessment of 50 cases after 3 months (Cohen’s κ). Interpretation followed Landis and Koch criteria.

### Propensity score matching

To reduce selection bias between hook plate fixation and locking plate fixation, propensity score matching was performed prior to comparative analysis. Patients were matched 1:1 based on age, sex, and AO fracture classification using nearest-neighbor matching without replacement. The resulting matched cohort was used for all subsequent comparisons.

### Statistical analysis

All statistical analyses were performed using Python and R (version 4.5.2). Categorical variables were compared using the chi-square test or Fisher’s exact test, and continuous variables using the t-test or Mann–Whitney U test, as appropriate. A p-value < 0.05 was considered statistically significant. Inter- and intra-rater reliability were assessed using Fleiss’ κ and Cohen’s κ and interpreted according to Landis and Koch.

## Results

### Patient demographics

After applying the inclusion and exclusion criteria, a total of 101 lateral clavicle fractures (Allman II / AO type 15-C) were identified. Among these, fixation was performed using a hook plate in 45 cases and locking plate fixation in 56 cases, including 8 cases in which additional interfragmentary screw fixation was used. After propensity score matching, 88 patients with lateral clavicle fractures (Allman II / AO type 15-C) were included, with 44 patients treated using a hook plate and 44 using locking plate fixation. Median follow-up time was 26 weeks (IQR 12–52 weeks; mean 33.0 ± 18.1 weeks; minimum 6 weeks), with 22 patients having follow-up exceeding 52 weeks. There were no significant differences between groups regarding age, sex, side of injury, polytrauma status (Injury Severity Score ≥ 16), or AO fracture classification. The distribution of fracture fragments was comparable between groups without statistically significant differences (Table 1).

### Operative time and complications

Mean operative time was significantly shorter in the hook plate group compared to the locking plate group (77.8 ± 28.4 vs. 93.5 ± 30.2 min; *p* = 0.018) (Table 2).

Overall complication rates were higher in the hook plate group compared to the locking plate group, although this difference did not reach statistical significance (18.2% vs. 4.5%, *p* = 0.093). The most frequent complications in the hook plate group were infection, osteolysis, and implant-related irritation, whereas complications after locking plate fixation were rare. One peri-implant fracture occurred in each group prior to implant removal, both occurring after subsequent trauma events (Table 3). All reported complications required reoperation.

### Implant removal and refracture

Implant removal was performed in all patients treated with a hook plate, whereas only a minority of patients in the locking plate group underwent implant removal (100% vs. 25%, *p* < 0.001). Time to implant removal was significantly shorter in the hook plate group compared to the locking plate group (6.7 ± 4.1 vs. 24.4 ± 10.5 months, *p* < 0.001). Among patients who underwent implant removal, the duration of the removal procedure was significantly shorter after hook plate fixation (37.7 ± 21.5 vs. 65.7 ± 40.3 min; *p* = 0.003). Refractures after implant removal occurred in 2 patients (4.5%) in the hook plate group and 1 patient (9.1%) in the locking plate group (*p* = 0.498); percentages refer to patients who underwent implant removal (Table 4).

### Callus formation

No significant difference in radiographic callus formation was observed between groups (*p* = 0.592). Early callus formation (≤ 6 weeks) occurred in 45.5% of patients in the hook plate group and 47.7% in the locking plate group, whereas absence of callus was uncommon in both groups (Table 5).

Inter- and intra-rater reliability for radiographic evaluation showed moderate agreement (Fleiss’ κ = 0.48; Cohen’s κ = 0.45).

## Discussion

Lateral clavicular fractures, representing up to 20% of all clavicular fractures, pose unique challenges during surgical intervention [1, 5]. As a possible solution to these challenges, hook plates were introduced [13]. Their clinical use, however, remains controversial, with variable functional outcomes and higher complication and revision rates reported compared to alternative fixation methods [20].

A critical gap in the existing literature is the lack of comprehensive analysis regarding operative efficiency between hook plates and alternative fixation methods. Given the increasing emphasis on healthcare economics, OR utilization, and patient safety considerations related to anesthesia exposure, understanding the true operative demands of different fixation strategies has become increasingly important [21, 22].

In this consecutive single-center cohort of 88 distal clavicle fractures, hook plates were associated with significantly shorter primary operative times than alternative plating strategies, without detectable differences in complication rates, refracture after removal, or radiographic callus timing. These results add an operative-efficiency perspective to a literature that has largely emphasized union and complications while seldom quantifying theatre time.

Our data showing an approximately 17% reduction in operative time with hook plates therefore adds new insight into their practical application. The shorter operative time observed with hook plates may be related to the comparatively straightforward surgical technique, which eliminates the need for complex reduction maneuvers, added screws and wires, and extensive soft tissue dissection often required with alternative fixation methods [23, 24]. The biomechanical principle underlying hook plate efficiency relates to the subacromial hook providing immediate stability, maintaining fracture reduction through the fulcrum effect beneath the acromion while screws are placed in the medial fragment [2].

A reduction of 16.7 min may have implications for operating room utilization. Although the present study was not designed as an economic analysis, the observed time difference may translate into reduced anesthesia duration and improved operating room turnover. From the perspective of the Swiss trauma system, OR time is a system-limiting resource rather than a financial metric [25]. These considerations should be regarded as hypothesis-generating, as the present study was not designed to evaluate economic outcomes or system-level effects.

Shorter operative duration may reduce anesthesia exposure, which has been associated with lower peri-anesthetic risk in previous studies, particularly in high-acuity trauma settings, although this was not specifically analyzed in the present cohort [25–27]. Although many lateral clavicle fractures are treated after initial stabilization rather than as part of emergency damage-control surgery, operative time may still be relevant in acute trauma situations where operating room resources are limited [25].

Previous meta-analytic data have also suggested an association between prolonged operative time and increased infection risk [28]. However, anesthesia-related complications, infection risk, and fatigue-related performance effects were not specifically assessed in the present study, precluding causal conclusions. Accordingly, shorter operative time should be considered primarily as a measure of perioperative efficiency rather than proven clinical benefit [29].

A critical aspect of hook plate fixation is the near-routine need for implant removal, which has been consistently reported in the literature [7, 30]. When cumulative operative time is considered (index procedure plus implant removal), hook plates resulted in longer total operative exposure compared with locking plate fixation in the present cohort.

This finding contrasts with the shorter operative time observed for the index procedure alone and should be considered when evaluating the overall surgical burden associated with each technique. Because implant removal represents a planned secondary procedure performed under different clinical circumstances, the relative relevance of index operative time versus cumulative operative exposure may vary depending on the clinical context [7, 30].

Prior comparative studies and meta-analyses report broadly similar union and functional outcomes between hook plates and other plates but often highlight implant-specific adverse events and the near-routine need for hardware removal with hook plates [31].

The systematic review by Shahbaz et al. also highlighted that complication rates for hook plates vary widely in the literature (8–32%) [30]. The observed complication rate in the present matched cohort (18.2%) falls within this reported range. Although no statistically significant difference in complication rates was detected between groups (18.2% vs. 4.5%, *p* = 0.093), the absolute complication rate of nearly 20% in the hook plate group remains clinically relevant and should be considered when selecting the surgical technique. However, the study was underpowered to detect differences in complication endpoints, and these findings should therefore be interpreted with caution. Accordingly, previous reports suggesting higher complication rates with hook plates [24, 32] can neither be confirmed nor excluded based on the present data. Refracture after implant removal was uncommon in both groups (4.5% vs. 9.1%, 2.3% vs. 6.2%) and consistent with previous literature describing refracture as an infrequent but multifactorial event after clavicle osteosynthesis [33]. 

Furthermore, no statistically significant difference in radiographic callus appearance was observed between groups. However, no dedicated radiographic parameters reflecting reduction quality or construct stability, such as coracoclavicular distance or secondary displacement, were systematically assessed. Callus formation was used as a surrogate radiographic parameter and does not necessarily reflect time to union or clinical recovery. Most previous studies on distal clavicle fixation have focused on union rates, complications, and functional outcomes rather than timing of callus formation, which limits direct comparability of this parameter [1, 7, 30, 34]. Therefore, the clinical relevance of the observed callus patterns should be interpreted with caution, particularly given the only moderate inter- and intra-rater agreement in the present analysis.

A possible explanation for the observed differences in callus appearance may relate to differences in construct stability; however, because callus formation represents only a surrogate parameter, no firm conclusions regarding fracture healing can be drawn from the present data. Future studies using validated union criteria and functional outcome measures are required.

### Limitations

This study has several limitations. Its retrospective single-center design introduces potential selection bias, as implant choice was not randomized and may have been influenced by surgeon preference, implant availability, fracture morphology, and temporal changes in surgical practice during the study period (2016–2024). Although propensity score matching was performed, residual confounding cannot be excluded and the comparative findings should therefore be interpreted as exploratory.

Callus formation was assessed using routine radiographs and represents a surrogate parameter that is not an established outcome measure for distal clavicle fixation. No dedicated radiographic parameters reflecting reduction quality or construct stability, such as coracoclavicular distance or secondary displacement, were assessed; although clinically relevant displacement would likely have been captured as a complication, more subtle differences cannot be excluded. Inter- and intra-rater reliability was only moderate, and no direct assessment of radiographic union, functional recovery, or patient-reported outcome measures (PROMs) was available; therefore, the clinical relevance of the callus analysis is limited. MRI was not routinely performed, and rotator cuff pathology related to hook plate fixation could not be systematically evaluated.

Operative time was defined as incision-to-closure and may not fully reflect differences in case complexity, particularly in polytrauma patients. Functional outcomes, PROMs, and range-of-motion data were not systematically available. The study was not powered to detect differences in complication rates, as no a priori sample size calculation was performed and the cohort size was determined by the available retrospective data. Accordingly, the clinical relevance of the observed differences should be interpreted with caution and primarily in the context of perioperative parameters.

Finally, the high proportion of polytrauma patients in this tertiary trauma center cohort may limit generalizability to lower-acuity settings. No dedicated subgroup analysis for polytrauma, emergency status, or perioperative risk scores (e.g., ASA, ISS, ICU stay) was performed, and the impact in high-acuity trauma settings was not specifically evaluated.

## Conclusion

In this propensity score–matched cohort of 88 AO/OTA 15-C distal clavicle fractures, hook plate fixation was associated with a shorter primary operative time compared with locking plate fixation, without statistically significant differences in complication rates, refracture after implant removal, or radiographic callus appearance.

Because hook plates require routine secondary implant removal, cumulative operative exposure may be greater despite the shorter index procedure. Implant selection should therefore be individualized, balancing intraoperative efficiency against the need for planned hardware removal.

As functional outcomes and patient-reported outcome measures (PROMs) were not available, the findings should be interpreted primarily in the context of perioperative parameters rather than clinical superiority. Further prospective studies including functional outcomes and PROMs are needed to better define the role of hook plates in distal clavicle fracture fixation.

**Table 1:** Patient demographics and fracture characteristics

Variable	Group HP (*n* = 44)	Group LCP (*n* = 44)	*p*-value
Age (years), mean ± SD	46.8 ± 16.3	49.0 ± 16.6	0.5320
Male, n (%)	33 (75.0%)	34 (77.3%)	1.0000
Right side, n (%)	22 (50.0%)	22 (50.0%)	1.0000
Polytrauma, n (%)	11 (25.0%)	9 (20.5%)	0.7990
AO (15-C1 n (%) / 15-C2 n (%))	32 (72.7%) / 12 (27.3%)	36 (81.8%) / 8 (18.2%)	0.4450
Fragment Count			0.0760
Two-part n (%)	18 (40.9%)	9 (20.5%)	
Three-part n (%)	15 (34.1%)	16 (36.4%)	
Multifragmentary n (%)	11 (25.0%)	19 (43.2%)	

**Table 2:** Operative time

Variable	Group HP (*n* = 44)	Group LCP (*n* = 44)	*p*-value
Operating time (min), mean ± SD	77.8 ± 28.4	93.5 ± 30.2	0.018

**Table 3:** Complications

Complications	Group HP (*n* = 44)	Group LCP (*n* = 44)	*p*-value
**No**	36 (81.8%)	42 (95.5%)	
**Yes**	**8 (18.2%)**	**2 (4.5%)**	**0.093**
Infection	2	0	
Osteolysis	2	0	
Material failure*	1	0	
Peri-implant fracture	1	1	
Pseudoarthrosis	1	1	
Pain	1	0	

* Material failure includes implant-related complications such as screw loosening, breakage, or loss of fixation

**Table 4:** Implant removal outcomes (patients with implant removal only)

Variable	Group HP (*n* = 44)	Group LCP (*n* = 11)	*p*-value
Duration implant removal (min), mean ± SD	37.7 ± 21.5	65.7 ± 40.3	0.003
Refracture after implant removal, n (%)	2 (4.5%)	1 (9.1%)	0.498

**Table 5:** Callus formation

Callus formation	Group HP (*n* = 44)	Group LCP (*n* = 44)	*p*-value
Early (≤ 6 w)	20 (45.5%)	21 (47.7%)	
Intermediate (> 6–26 w)	21 (47.7%)	22 (50.0%)	
None	3 (6.8%)	1 (2.3%)	0.592

## Data Availability

No datasets were generated or analysed during the current study.
